# Perioperative prophylaxis with ertapenem reduced infections caused by extended-spectrum betalactamase-producting Enterobacteriaceae after kidney transplantation

**DOI:** 10.1186/s12882-019-1461-4

**Published:** 2019-07-22

**Authors:** Gemma Sanclemente, Marta Bodro, Carlos Cervera, Laura Linares, Frederic Cofán, Francesc Marco, Jordi Bosch, Federico Oppenheimer, Fritz Dieckmann, Asunción Moreno

**Affiliations:** 1 Department of Infectious Diseases, Hospital Clinic – IDIBAPS, University of Barcelona, Villarroel 170, 08036 Barcelona, Spain; 2 Kidney Transplant Unit, Hospital Clinic – IDIBAPS, University of Barcelona, Barcelona, Spain; 3 Department of Microbiology, Centre Diagnòstic Biomèdic (CDB), Instituto de Salud Global de Barcelona (ISGlobal), Hospital Clinic – IDIBAPS, University of Barcelona, Barcelona, Spain

**Keywords:** Kidney transplantation, Infection, Surgical prophylaxis, Multidrug-resistant bacteria

## Abstract

**Backgound:**

In recent years we have witnessed an increase in infections due to multidrug-resistant organisms in kidney transplant recipients (KTR). In our setting, we have observed a dramatic increase in infections caused by extended-spectrum betalactamase-producing (ESBL) *Enterobacteriaceae* in KTR. In 2014 we changed surgical prophylaxis from Cefazolin 2 g to Ertapenem 1 g.

**Methods:**

We compared bacterial infections and their resistance phenotype during the first post-transplant month with an historical cohort collected during 2013 that had received Cefazolin.

**Results:**

During the study period 110 patients received prophylaxis with Cefazolin and 113 with Ertapenem. In the Ertapenem cohort we observed a non-statistically significant decrease in the percentage of early bacterial infection from 57 to 47%, with urine being the most frequent source in both. The frequency of infections caused by *Enterobacteriaceae* spp. decreased from 64% in the Cefazolin cohort to 36% in the Ertapenem cohort (*p* = 0.005). In addition, percentage of ESBL-producing strains decreased from 21 to 8% of all *Enterobacteriaceae* isolated (*p* = 0.015). After adjusted in multivariate Cox regression analysis, male sex (HR 0.16, 95%CI: 0.03–0.75), cefazolin prophylaxis (HR 4.7, 95% CI: 1.1–22.6) and acute rejection (HR 14.5, 95% CI: 1.3–162) were associated to ESBL- producing *Enterobacteriaceae* infection.

**Conclusions:**

Perioperative antimicrobial prophylaxis with a single dose of Ertapenem in kidney transplant recipients reduced the incidence of early infections due to ESBL-producing *Enterobacteriaceae* without increasing the incidence of other multidrug-resistant microorganisms or *C*. *difficile*.

## Background

Infections are a major complication after kidney transplantation (KT). During the first post-transplant month, the majority of infections are caused by bacteria, most of them originating from the urine [1]. In recent years we have observed an increase in the incidence of infections caused by multidrug-resistant microorganisms, especially ESBL-producing *Enterobacteriaceae* [2]. These infections have been associated not only with increased costs, but also with higher mortality and graft loss [3, 4]. Perioperative prophylaxis is administered to prevent surgical site infections but, in the case of urological procedures, it also helps prevent postoperative bacteriuria. Classically guidelines recommend a single dose of Cefazolin in clean-contaminated surgical procedures, as is the case for kidney transplantation [5]. In our centre we have observed a high incidence of early infections caused by ESBL *Enterobacteriaceae;* the prevalence of infections caused by ESBL-producing *Enterobacteriaceae* in 2012 in kidney transplant recipients was 12%, mainly urinary tract infections (80%). For this reason, and based on the published data on the efficacy and safety of Ertapenem for surgical prophylaxis [6], we decided to change the antimicrobial prophylaxis for KT patients from Cefazolin 2 g to Ertapenem 1 g.

The aim of this study was to compare the incidence and susceptibility profile of bacterial infections in the first month after KT between patients who received Cefazolin and those who received Ertapenem.

## Methods

We conducted an observational study at a tertiary university referral hospital with an active kidney transplantation programme (annual average of 120 procedures) in Barcelona, Spain. Until December 2013 all kidney transplant patients received a single dose of Cefazolin 2 g as perioperative antimicrobial prophylaxis. From January 2014 all patients undergoing KT received a single dose of Ertapenem 1 g. Although ertapenem requires a scaled dose adjustment in renal dysfunction in case of treatment, we do not consider adjustment because perioperative antimicrobial prophylaxis consists in a single dose of antibiotic. We collected data on all bacterial infections that occurred during the first post-transplant month, and compared patients who received a KT during 2013 (Cefazolin group, historical cohort) and patients undergoing KT during 2014 (Ertapenem group). Data was prospectively recorded from January to December 2014 and data from the historical cohort was collected retrospectively. Patients who received other perioperative antimicrobial prophylaxis were excluded. Cotrimoxazole was prescribed in all recipients for the prevention of *Pneumocystis jirovecii* pneumonia, given from the first day of oral tolerance until the sixth month post-transplantation. Double transplants were excluded. During the first month after transplantation follow-up was performed weekly. We routinely collect urine cultures after urinary catheter removal. Ureteral stents were only used in orthotopic transplantation (< 5% of all proceedings). Eighty-one kidney recipients received the monoclonal anti-IL2 receptor antagonist basiliximab therapy and 115 received rabbit anti-thymocyte globulin (ATG) as induction therapy. All patients received Corticosteroids and dose was progressively decreased from initially 1 mg/kg/day to 5 mg/day at 3 months post-transplantation. Mycophenolate mofetil (MMF) or sirolimus with tacrolimus or cyclosporine were maintenance immunosupression.

### Definitions

Urinary tract infection (UTI) was diagnosed based on the guidelines of the European Society of Clinical Microbiology and Infectious Disease Infectious Diseases Society of America [7, 8] and Guidelines from the American Society of Transplantation Infectious Diseases Community of Practice [9]. Asymptomatic bacteriuria was defined when more than 100.000 UFC/mL of urinary pathogens were found in aseptically collected midstream urine in absence of symptomatology. Acute uncomplicated UTI (including cystitis and prostatitis) was defined when recipients presented urinary frequency/urgency, dysuria, suprapubic pain but no indwelling device and no systemic symptoms such as fever, allograft pain or hemodynamic compromise were present, and a urine culture yielding growth of more than 100.000 CFU/mL of urinary pathogens. Complicated UTI, including acute graft pyelonephritis or upper tract UTI, was defined as at least one of the following: malaise, chills, fever, hemodynamic instability, leukocytosis, pain over the allograft or the costovertebral angles for allograft or native kidney involvement, bacteremia with the same organism identified in urine culture and a significant growth of a uropathogen (≥10.000 CFU/mL).

Surgical site infection (SSI) was defined as those involving only skin and incisional subcutaneous tissue. Deep incisional SSI was present when involving deep tissues, including also infections draining through incision. Organ/space SSI was considered if involving any part of the anatomy in organs and spaces manipulated during transplant surgery [10].

Venous catheter-related bloodstream infection was defined as the presence of bacteremia originating from an intravenous catheter when documenting a blood isolated cultured from the catheter tip using the Maki’s semiquantitative rollplate catheter culture (≥ 15 CFU). Primary or unknown source bacteremia was considered when a one or more blood cultures and organism cultured from blood was not related to an infection at another site.

Patients with septic shock can be identified by presenting a systolic pressure < 90 mmHg that was unresponsive to fluid therapy or required vasoactive drug treatment.

All patients diagnosed of acute allograft rejection had biopsy. If kidney recipients required definitive hemodialysis, graft loss was considered.

We used Magiorakos et al. [11] criteria to defined multidrug resistance (MDR). Briefly, we considered *Enterobacteriaceae* and *Pseudomonas aeruginosa* to be MDR when a strain was resistant to one or more agent in three or more antimicrobial categories normally active against the isolated bacteria. For *S. aureus*, methicillin-resistant strains were considered MDR.

### Microbiological studies

Matrix-Assisted Laser Desorption/Ionization Time of Flight (MALDI-TOF) technique was performed to identify microorganisms. Susceptibility testing of microorganisms recovered was done using the Phoenix automated system (Becton Dickinson Company, Sparks, Maryland), E-test or Kirby-Bauer disc-diffusion methods. To define susceptibility or resistance to antimicrobial agents we used the criteria of the European Committee on Antimicrobial Susceptibility Testing (EUCAST) available at the time of diagnosis. Extended spectrum beta-lactamases (ESBLs) are defined as enzymes produced by certain bacteria that are able to hydrolyze extended spectrum cephalosporin and aztreonam (but not the cephamycins or carbapenems) and which are inhibited by β-lactamase inhibitors such as clavulanic acid [12]. EUCAST guidelines were followed in case of ESBL or a carbapenemase production suspiction [13, 14].

### Statistical analysis

We used SPSS statistical package (version 18.0; SPSS, Chicago, Illinois, USA) to perform statistical analysis, using the *χ*^*2*^ or Fischer exact test when comparing categorical variables and the Student *t* test or non-parametric tests depending on the homogeneity of the variable to compare continuous variables. We used Kaplan-Meier method to perform survival curves. We assessed the impact of age, sex, prior transplantation, prophylaxis group, reoperation, acute allograft rejection, diabetes mellitus and post transplant hemodyalisis requirement on presenting infection caused by ESBL-producing *Enterobacteriaceae* using Cox proportional hazards regression model to calculate hazard ratios (HRs) and 95% confidence intervals (CIs). All statistical tests were two-tailed, and the threshold of statistical significance was set at *p* < 0.05.

## Results

During the study period, 110 patients received prophylaxis with Cefazolin and 113 with Ertapenem. We found no differences in the baseline pre-transplant variables, immunosuppression, non-infectious post-transplant complications or the incidence of early infection between cohorts (Table 1).

**Table 1:** Clinical characteristics of the cohort according to prophylaxis received

Variable	Cefazolin (*n* = 110)	Ertapenem (*n* = 113)	*P*
Age in years (mean, ±SD)	54.02 (13.6)	53.99 (14.7)	1
Male sex	61 (55%)	65 (57%)	0.7
Donor type			
Deceased	53 (48%)	61 (54%)	0.4
Live	57 (52%)	52 (46%)	
Donor’s cause of death			
Anoxia	14 (26%)	8 (15%)	0.1
CVA	31 (58%)	42 (80%)	
Trauma	7 (13%)	2 (4%)	
Other	1 (2%)	0	
Median ischemia time (minutes, ±SD)	473 (470)	491 (434)	0.4
Diabetes mellitus	24 (22%)	26 (23%)	0.8
End-stage renal disease			
Glomerulonephritis	9 (8%)	6 (5%)	0.9
Diabetes mellitus	17 (16%)	17 (16%)	
Hypertension	19 (17%)	16 (14%)	
Cystic kidney disease	18 (16%)	17 (15%)	
Other Urologic	8 (7%)	10 (9%)	
Other cause	23 (21%)	23 (20%)	
Unkown/missing	16 (15%)	24 (21%)	
Prior transplantation	18 (16%)	22 (19%)	0.6
Immunosuppression regimen			
CNI + MMF+ CS	72 (65%)	64 (57%)	0.3
CNI + mTOR+ CS	34 (31%)	46 (41%)	
Other	4 (4%)	3 (2%)	
Induction			
None	18 (16%)	9 (8%)	0.4
Basiliximab	26 (24%)	55 (49%)	
Anti-lymphocyte globulines	66 (60%)	49 (43%)	
Pre-transplant rituximab	13 (12%)	13 (11%)	0.9

*CVA* Cerebrovascular accident, *CNI* Calcineurin inhibitors, *MMF* Mycophenolate mofetil, *mTOR* Inhibitors of mammalian target of rapamycin, *CS* Corticosteroids

Outcomes are described in Table 2. Sixty-three patients in the Cefazolin group (57%) developed at least one episode of bacterial infection during the first month after transplantation compared to 53 patients (47%) in the Ertapenem group (*p* = 0.1). Ten patients of the Cefazolin group and 11 in the Ertapenem group presented two or more episodes of bacterial infection respectively. When we analysed only clinically significant infections (excluding asymptomatic bacteriuria from the analysis), the incidence was similar in both cohorts (26% in those who received Cefazolin and 20% in those who received Ertapenem, *p* = 0.2). Median days until urinary catheter removal were 9 (IQR 4–48).

**Table 2:** Outcomes of patients depending on perioperative antibiotic prophylaxis

Variable	Cefazolin (*n* = 110)	Ertapenem (*n* = 113)	*P*
Post-transplant complications (first month)			
Acute rejection	21 (19%)	15 (13%)	0.2
Haemodialysis	18 (16%)	25 (22%)	0.3
Reoperation	14 (13%)	12 (11%)	0.4
Nephrostomy	6 (5%)	6 (5%)	1
Ureteral stent	4 (4%)	11 (10%)	0.06
Days of urinary catheter removal (mean, SD)	9 (6)	9 (6)	0.8
Patients with infection (first month)	63 (57%)	53 (47%)	0.1
Patients with clinically significant infection (first month)^a^	29 (26%)	22 (20%)	0.2
Days until first infection (mean, SD)	10 (7)	11 (7)	0.7
Graft lost (30 days)	0	2 (2%)	1
Mortality (30 days)	0	1 (1%)	0.9

^a^Clinically significant infection: excluding asymptomatic bacteriuria

If excluding asymptomatic bacteriuria, the timeline to the occurrence of a first infection after transplantation did not differ between groups (mean 10 days). The main source of infection was the urinary tract in both groups (85 and 70% in the Cefazolin and Ertapenem groups respectively, *p* = 0.09). Ten episodes (14%) in the Cefazolin group and eight (12%) in the Ertapenem group had positive blood cultures (*p* = 0.43). Regarding microbiology of bacteremic episodes, 28% were caused by *P. aeruginosa*, followed by *E.coli* (22%), *K. pneumonia*e (22%) and Staphylococci (11%). Regarding bacteremic episodes, the most important source of infection was urinary in the Cefalozin group (6 patients) and the venous catheter (4 patients) in the Ertapenem group respectively. Moreover, two episodes of bacteremia in the Cefazolin group and none in the Ertapenem group were produced by MDR organisms. There was no difference between the two groups in terms of post-transplant complications, graft lost or mortality at 30 days.

Regarding infection foci, 34% of episodes of urinary tract infections were caused by *E.coli, followed by Enterococcus* spp. (34%), *P. aeruginosa* (7%) and *K. pneumoniae* (1%). Main aetiologies of SSI were *Enterococcus* spp. (34%), *E.coli* (33%) and *S. aureus* (22%).

We also performed a subanalysis of patients presenting with an ESBL-producing *Enterobacteriaceae* infection the first month after transplantation. All patients except 4 had urinary cultures within 2 months before transplantation (in case of residual diuresis). None of them had presented an infection caused by ESBL-producing *Enterobacteriaceae* prior transplantation.

We found no differences between groups regarding the incidence of infections caused by *Pseudomonas aeruginosa* (12% vs 14%, *p* = 0.9), *Enterococcus* spp. (33% vs 47%, *p* = 0.1), *Candida* spp. (9% vs 5%, *p* = 0.1) or *Clostridium difficile* (1% vs 2%, *p* = 1). *E. faecium* was isolated more frequently in the Cefazolin group (54%) than in the Ertapenem group (26%, *p* = 0.03), but none of the isolates were resistant to vancomycin. We did not detect any carbapenem-resistant *Enterobacteriaceae*. All episodes of Candida spp. infections were UTI. Only one episode of candidemia was diagnosed during the study period (in the group of ertapenem prophylaxis). Table 3 summarizes the characteristics of the infectious episodes.

**Table 3:** Differences in clinical and microbiological characteristics of infectious episodes between the two cohorts

Variable	Cefazolin (*n* = 73)	Ertapenem (*n* = 67)	*P*
Source of infection			
Urinary	62 (85%)	47 (70%)	0.09
SSI	3 (4%)	6 (9%)	0.5
Other	8 (11%)	14 (21%)	0.3
Positive blood cultures	10 (14%)	8 (12%)	0.4
Septic shock	2 (3%)	1 (2%)	0.6
Isolated microorganisms			
*Enterobacteriaceae*	47 (64%)	24 (36%)	0.005
ESBL-producing	10 (21%)^a^	2 (8%)^b^	0.01
*P. aeruginosa*	9 (12%)	9 (14%)	1
XDR	5 (56%)	2 (22%)	0.2
*Enterococcus* spp.	24 (33%)	31 (47%)	0.1
*E. faecium*	13 (54%)	8 (26%)	0.03
*C. difficile* colitis	1 (1%)	2 (3%)	1
*Candida spp.* infection	7 (9%)	3 (5%)	0.1
CR *Enterobacteriaceae*	0	0	

*ESBL* Extended-spectrum betalactamase-producing, *XDR* Extensively drug-resistant, *CR* Carbapenem-resistant

^a^seven episodes were due to *Klebsiella pneumoniae* and three to *E.coli*

^b^all episodes were due to *Klebsiella pneumoniae*

Multivariate cox regression analysis to evaluate risk for ESBL-producing *Enterobacteriaceae* infection among kidney recipients depending on some variables was performed in Table 4. According to HR figures, male sex (HR 0.16, 95% CI: 0.03–0.75), cefazolin prophylaxis (HR 4.7, 95% CI: 1.1–22.6) and acute allograft rejection (HR 14.5, 95% CI: 1.3–162) were associated to ESBL- producing *Enterobacteriaceae* infection. Nevertheless, age < 50 years (HR 0.5, 95% CI: 0.1–2.7), diabetes mellitus (HR 0.9, 95% CI: 1.6–5.7), post-transplant haemodyalisis (HR 0.3, 95% CI: 0.06–1.2), nephrostomy requirement (HR 0.8, 95% CI: 1.3–0.1) and reoperation (HR 2.6, 95% CI: 0.6–12) could not be considered risk factors for ESBL-producing Enterobacteriaceae infection.

**Table 4:** Multivariate cox regression analysis of risk factors for ESBL-producing *Enterobacteriaceae* infection among kidney recipients depending on some variables

Variable	ESLB-producing *Enterobacteriaceae* infection		HR (95%CI)	*P*
	Yes	No		
Age				
< 50 years	2 (17%)		0.5 (0.1–2.7)	0.5
≥ 50 years	10 (83%)	62 (60%)		
Sex				
Male	2 (17%)	58 (56%)	0.16 (0.03–0.75)	0.02
Female	10 (83%)	46 (44%)		
Diabetes mellitus				
Yes	2 (17%)	80 (77%)	0.9 (1.6–5.7)	0.9
No	10 (83%)	24 (23%)		
Prior transplantation				
Yes	3 (25%)	86 (83%)	5.8 (1.2–30)	0.04
No	9 (75%)	18 (17%)		
Prophylaxis group				
Cefazolin	10 (83%)	53 (51%)	4.7 (1.1–22.6)	0.05
Ertapenem	2 (17%)	51 (49%)		
Acute allograft rejection				
Yes	1 (8%)	26 (25%)	14.5 (1.3–162)	0.03
No	11 (92%)	78 (75%)		
Post-transplant Haemodialysis				
Yes	3 (25%)	26 (25%)	0.3 (0.06–1.2)	0.09
No	9 (75%)	78 (75%)		
Nephrostomy				
Yes	1 (8%)	8 (8%)	0.8 (1.3–0.1)	0.8
No	11 (92%)	96 (92%)		
Reoperation				
Yes	3 (25%)	11 (11%)	2.6 (0.6–12)	0.2
No	9 (75%)	93 (89%)		

Regarding the aetiology of infectious episodes, we observed a significantly higher number of episodes caused by *Enterobacteriaceae spp.* in the Cefazolin group (47 episodes, 64% of all isolates) than in the Ertapenem group (24 epidodes, 36%) (*p* = 0.005). In addition, a higher percentage of isolates of *Enterobacteriaceae spp.* were ESBL-producers in the Cefazolin group (10 episodes, 21%) comparing with the Ertapenem cohort (2 episodes, 8%, *p* = 0.01).

The occurrence of ESBL-producing *Enterobacteriaceae* infections was not related to an active outbreak of nosocomial infection.

**Fig. 1:**
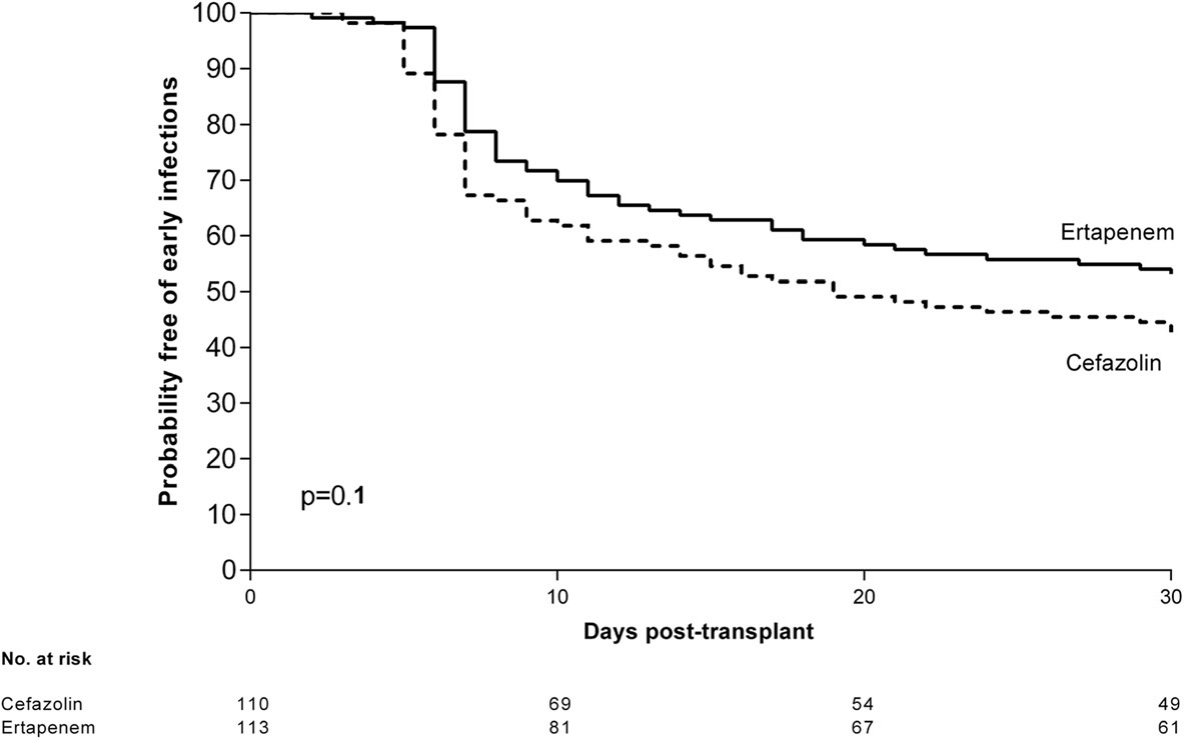
Kaplan–Meier survival graph for probability of early infections (first post transplantation month) by perioperative antibiotic prophylaxis. Patients with Ertapenem prophylaxis presented fewer early infections (47%) than those with Cefazolin prophylaxis (57%) but not reaching statistical significance (log-rank, *p* = 0.1)

**Fig. 2:**
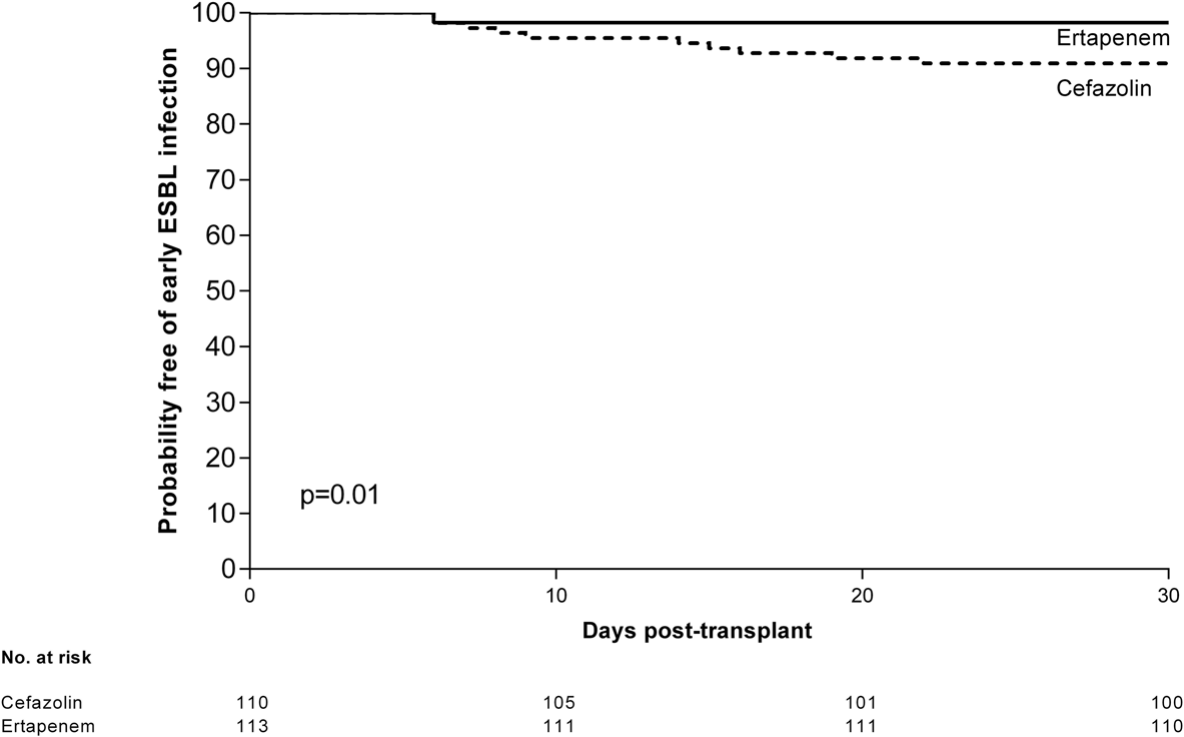
Kaplan–Meier survival graph for probability of ESBL- producing *Enterobacteriaceae* infection (first post transplantation month) by perioperative antibiotic prophylaxis. Patients with Ertapenem prophylaxis presented fewer infections caused by ESBL-producing *Enterobacteriaceae* (8%) than those with Cefazolin prophylaxis (21%) (log-rank, *p* = 0.01)

Figures 1 and 2 shows the Kaplan-Meier curves for probability of early infections and ESBL-producing Enterobacteriaceae infections respectively, by antibiotic prophylaxis received. Patients with Ertapenem prophylaxis presented fewer early infections (47%) than those with Cefazolin prophylaxis (57%) but not reaching statistical significance (log-rank, *p* = 0.1). Patients with Ertapenem prophylaxis presented fewer infections caused by ESBL-producing *Enterobacteriaceae* (8%) than those with Cefazolin prophylaxis (21%) (log-rank, *p* = 0.01).

None of the infections could be considered a donor-derived infection.

Graft loss at a 2-years follow-up was 4 and 7% between cefazolin and ertapenem group respectively (*p* = 0.2). Mortality at a 2-years follow-up was 7 and 4% comparing cefazolin and ertapenem group respectively (*p* = 0.4).

## Discussion

In this large cohort of adult kidney transplant recipients, we found that perioperative antimicrobial prophylaxis with a single dose of Ertapenem reduced the incidence by almost half of *Enterobacteriaceae* infections and, more importantly, that the incidence of ESBL-producing strains decreased significantly by a third compared to the use of a single dose of Cefazolin in the first moth post transplantation.

The main goal of perioperative prophylaxis is to reduce surgical site infections. According to the recent American guidelines on antimicrobial surgical prophylaxis, KTR should receive a single dose of Cefazolin 2g^5^. However, a study performed during the 1990s found no differences in the incidence of early infection in KT patients who received perioperative prophylaxis and those who did not, suggesting that surgical prophylaxis can be avoided in KT patients [15]. More recently, some authors suggested using prophylaxis only in patients with a higher risk of surgical site infection, such as recipients older than 65 years or with a body mass index higher than 35 [16]. Regardless of all of these considerations, in our cohort of KT recipients the incidence of surgical site infection was 4%, and was not the main route of early infection. Instead, UTI were the most frequent type of infection, as in many previous studies [17, 18].

Interestingly, we observed a trend towards a lower incidence of infections during the first month post-transplantation using a single dose of Ertapenem, especially UTI. Furthermore, we observed a significant reduction in the incidence of infections due to *Enterobacteriaceae* and, more importantly, those strains producing ESBL. It is well known that ESBL-producing Gram-negative enteric bacilli infections after KT are associated with a worse prognosis for both the graft and patient and a high risk of UTI recurrence [4, 19]. Similar to our results, a Brazilian study described a reduction in the incidence of early UTI after KT when adding gentamycin to the usual prophylaxis [20]. However, the use of aminoglycosides in the early period after KT is not desirable due to its potential nephrotoxicity. In contrast, prolonging the duration of prophylaxis seems to have no impact on the occurrence of surgical site infection and UTI [21]. Moreover, Ertapenem is efficacious and safe for the prophylaxis of patients with abdominal surgery including colorectal manipulation [6, 22, 23]. Bora et al. [24] recommend tacrolimus concentration monitoring and dose reductions when the two drugs are administered in combination. Nevertheless perioperative prophylaxis consists in a single dose of ertapenem before surgery and patients usually start tacrolimus 24 h after surgery, so we think that adjustments may not be necessary. To the best of our knowledge, this is the first study analysing the efficacy of Ertapenem for the surgical prophylaxis of KT recipients.

Other variables associated with infections caused by ESBL-producing *Enterobacteriaceae* were prior transplantation, acute allograft rejection and female gender. It has been reported that the relative faecal abundance of ESBL *E. coli* is associated with UTI in women who have not been exposed to antibiotics [25]. Prior transplantation and acute allograft rejection may act as surrogate markers for other variables that might increase the probability of colonization by these organisms, such as antibiotic exposure and health care relationship, as others have found [26, 27], or even reflect a overimmunosuppression state that favors infection. Most studies agree that the *Enterobacteriaceae* causing UTI are ascending infections coming from the bowel after a previous colonization. In the setting of transplantation, Bert et al. found that pre-transplant faecal carriage of ESBL *Enterobacteriaceae* was an independent risk factor for infections caused by these organisms after liver transplantation [28], while a reduction in infections caused by Gram-negative bacteria was documented after selective bowel decontamination [29].

The main concern over administering broad-spectrum antibiotics is the development of infections caused by drug-resistant organisms. However, data about antimicrobial resistance in *Pseudomonas aeruginosa* infections showed a significant increase of antimicrobial resistance at 3 days of antibiotic administration [30, 31]. Likewise, we previously reported that one of the risk factors for infections with ESBL enteric bacilli in KT recipients was the prescription of antibiotics in addition to habitual prophylaxis [32]. Although Itani et al. reported a higher incidence of *C. difficile* infection in patients submitted to colorectal surgery who received prophylaxis with Ertapenem [6], we found no evidence of an increase in *C. difficile* infection, *P. aeruginosa* or carbapenem-resistant *Enterobacteriaceae*. A recent surveillance study also reported that the use of Ertapenem is not associated with an increase in drug-resistant Gram-negative bacilli [33].

A surprising result of our study was the decline in the occurrence of *E. faecium* infections in the Ertapenem cohort. It is well known that the activity of Ertapenem against *Enterococcus faecalis* is marginal and that *E. faecium* is resistant to all betalactams [34]. However, some years ago Mainardi et al. reported that imipenem could inhibit the synthesis of *E. faecium* peptidoglycan [35]. More recently, Dubée et al. reported this property for Ertapenem although its activity is lower than imipenem [36]. These studies analysed only the molecular basis of these interactions, but no study has evaluated its impact in the clinical setting. Nevertheless we hypothesize that the lower incidence of *E. faecium* and *P. aeruginosa* in the Ertapenem group were either random events or possibly due to an unknown factor not included in the analysis.

In recent years we have observed in our kidney transplant unit an increase in the incidence of infections caused by multidrug-resistant microorganisms, especially ESBL-producing *Enterobacteriaceae* and similar data has published in other centres worldwide [37–39]. Infections caused by multidrug-resistant pathogens caused an increasing number of healthcare-associated infections, causing a significant increment in costs and morbidity and mortality and are often associated with ICU admission and prior antibiotic use [40].

Reducing *Enterobacteriaceae* infections, especially ESBL-producing strains could mean reducing hospitalization and costs. However, in a long-term analysis, there was no statistical difference in 2-year graft loss neither 2-year mortality between the groups of prophylaxis. To avoid infection and especially colonization due to drug-resistant organisms some strategies have been described. First of all, shortening antibiotic regimens so as to decrease antibiotic-related selective pressures could be important prophylactic steps. Specifically, European guidelines recommend educational programmes based on hand hygiene, environmental cleaning, contact precautions and antimicrobial stewardship to reduce the horizontal spread of multidrug-resistant organisms during hospitalisation [41].

Our study had several limitations. First, as it was conducted in a single hospital ward, the results may not be applicable to other centres with different epidemiological backgrounds. Second, we did not perform a study of faecal carriage at the time of transplantation and, thus, we cannot rule out the possibility that the differences in the incidence of infections with *Enterobacteriaceae* between groups were due to different rates of pre-transplant bowel colonization. Furthermore, the combination of retrospective and prospective assessments limits the ability to truly compare the groups as it fails to consider the potential for local confounders in practices and epidemiologic changes. Moreover, we have no information about prior antibiotic exposure before transplantation. Although there were no changes in infection control protocol neither in surgical techniques or pre-transplant management during the study period, there could be potential pitfalls inherent in a comparison of two different eras. So, this data should be used to inform future more rigorous studies, including randomized ones.

## Conclusion

In conclusion, a single perioperative prophylactic dose of Ertapenem in KT was effective at preventing surgical site infection and decreased the incidence of infections due to *Enterobacteriaceae* during the first post-transplant month, with a particular impact on ESBL-producing strains and *E. faecium*. The use of Ertapenem did not increase the incidence of other drug-resistant microorganisms as *P. aeruginosa*, *C. difficile, Candida* spp*.* or carbapenem-resistant *Enterobacteriaceae*.

## Data Availability

Clinical data were prospectively or retrospectively recorded depending on the time period and introduced into a database with coded names to maintain anonymity.

## Abbreviations

ESBL: Extended-spectrum betalactamase-producing
KT: Kidney transplantation
KTR: Kidney transplant recipients
MDR: Multidrug-resistant
UTI: Urinary tract infection
